# Incidental finding of cardiac hydatid cysts, report of two cases

**DOI:** 10.1186/s12880-018-0268-2

**Published:** 2018-08-13

**Authors:** Dunya Moghul, Hidayatullah Hamidi

**Affiliations:** 1 Department of Pediatric Surgery, French Medical Institute for Mothers and Children (FMIC), Kabul, Afghanistan; 2 Department of Radiology, French Medical Institute For Mothers and Children (FMIC), Kabul, Afghanistan

**Keywords:** Case report, Hydatid cyst, Cardiac cyst, Echinococcus infection, Cardiac benign lesion

## Abstract

**Background:**

Hydatid is a parasitic infection which can affect any organ of body. In some organs like liver and lung; it can be found regularly while in other organs like heart, it is seen very rarely. Cardiac hydatid cysts comprise less than of 2% of hydatid infection cases and may be detected incidentally.

**Case presentation:**

Authors report two cases of cardiac hydatid cysts in young adult patients living in rural areas of the country with positive animal contact. Both patients were complained from shortness of breath and cough. Contrast enhanced chest computed tomography (CT) revealed left ventricular wall hydatid cysts in addition to lung and liver hydatid cysts.

**Conclusion:**

Cardiac hydatid cyst is a rare finding with wide range of signs and symptoms. These may be suspected in patients coming from endemic areas. Echocardiographic follow up of patients with liver or lung hydatid cysts can be helpful.

## Background

Echinococcus granulosus is one of the parasitic infections which can affect human beings. it is a common problem in the developed and developing countries specially in sheep raising areas [1]. Formation of cyst by this parasite in cardiac muscle is very rare and is reported in about 0.02–2% of cases [1, 2]. Authors report two cases of incidentally detected cardiac hydatid cysts in patients who underwent computed tomography study at authors’ institution.

## Case presentation

### Case 1

**Fig. 1:**
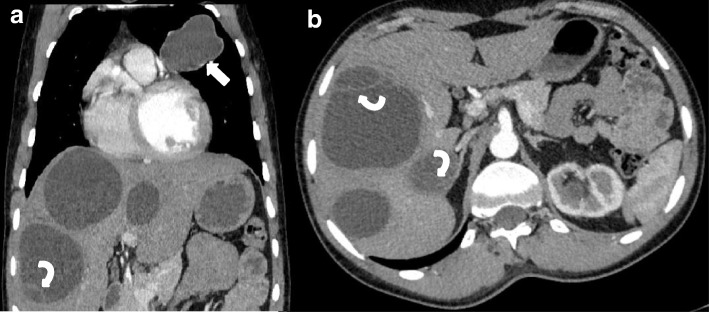
**a-b**: Contrast enhanced chest and upper abdominal CT coronal and axial sections: A well-defined, thin walled, low attenuating, cystic lesion with lobulated outlines in the anterior segment of the left upper lobe (arrow) and at least three cystic lesion in the liver one of them showing internal membrane (Curved arrows)

**Fig. 2:**
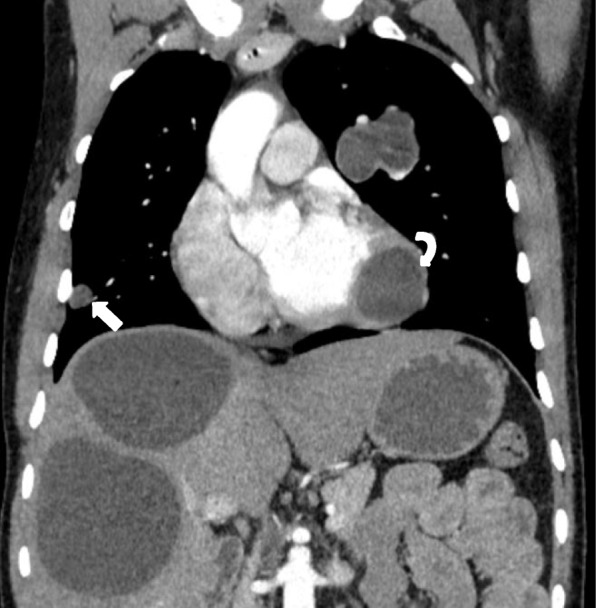
Contrast enhanced chest and upper abdominal CT coronal section: In addition the previously seen lesions in left lung and liver, a small pleural based cystic lesion in the right lung (arrow) as well as a larger lesion in the cardiac apex are seen (Curved arrow)

**Fig. 3:**
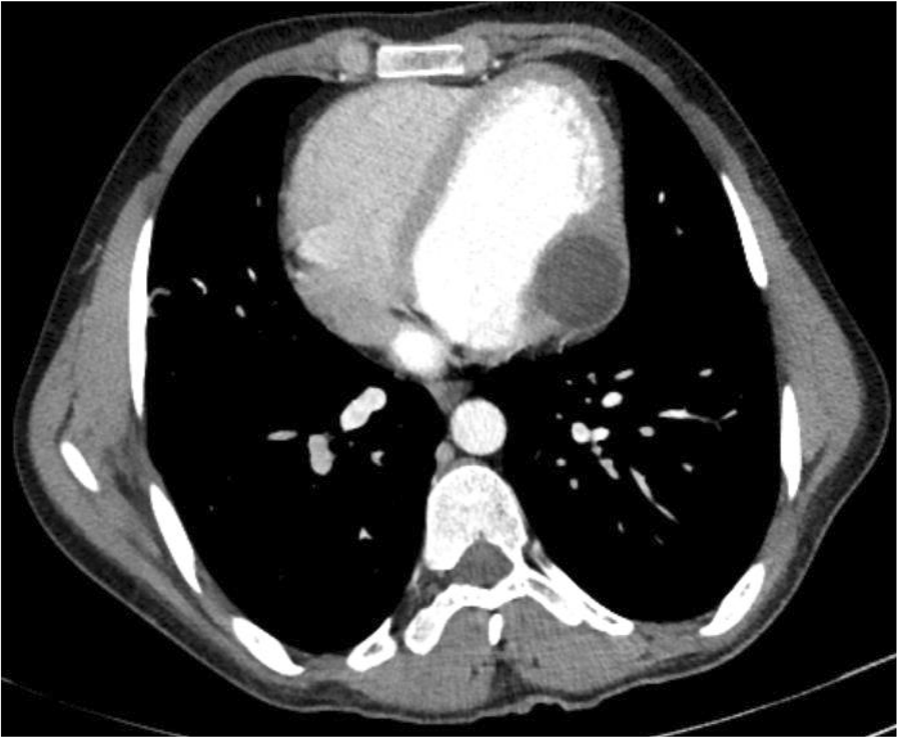
Contrast enhanced CT axial section through the heart: The cystic lesion in the heart is located in the lateral wall of the left ventricle

The first patient was a 20-year-old man keeping cows and sheep in house, living in a rural area of southern part of Afghanistan. The patient was complaining from shortness of breath and cough for last one and half year and was referred to undergo chest CT examination. A Contrast enhanced chest CT (with intravenous administration of 80 ml of non-ionic water soluble contrast material mnipaque-350-) revealed a well-defined, thin walled, low attenuating, cystic lesion with lobulated outlines in the anterior segment of the left lung upper lobe, measuring approximately 4.2 × 5.5 × 4.5 cm in size (Fig. 1a, arrow). Another small (1.5 × 1.5 cm) cystic lesion with same characteristics was seen in the anterior segment of right lung upper lobe (Fig. 2- arrow). A lesion of same characteristics was seen in the lateral wall of left ventricle which measured 3.6 × 3.9 × 3.5 cm (Fig. 2- curved arrow and Fig. 3).

Imaged sections through the abdominal cavity revealed at least seven cystic lesions in the liver. The largest lesion in segment seven of liver measured 7 × 8 cm. Some of the lesions demonstrated internal detached membrane, the so called water lily sign (Fig. 1a and b, curved arrows). No solid enhancing components, wall calcification or adjacent infiltrative/inflammatory changes were noted with these lesions.

### Case 2

**Fig. 4:**
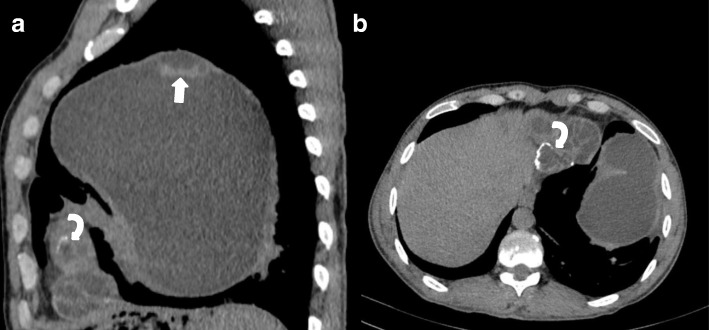
**a-b**: Unenhanced chest CT sagittal and axial sections: a large, well defined, fluid attenuating, non-enhancing cystic mass lesion in the left lung mostly occupying the left lung upper lobe. Some internal septations are seen in the superior aspect of this cystic lesion (Arrow). Parts of a multi-Septate, fluid attenuating cystic lesion is also seen in the cardiac apex with peripheral wall calcification (curved arrow)

**Fig. 5:**
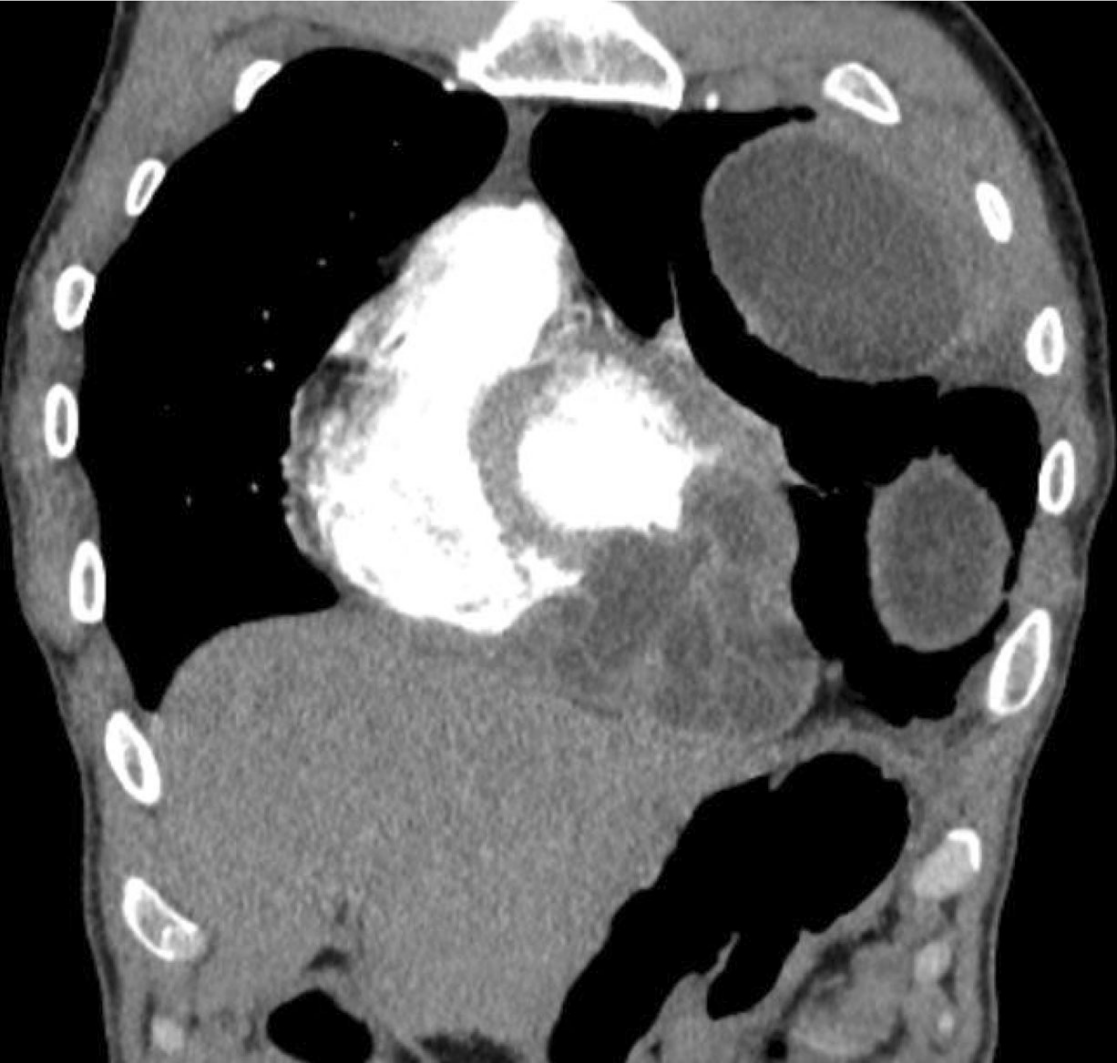
Contrast enhanced chest CT, coronal section: A multi-Septate, fluid attenuating cystic lesion in the cardiac apex

A 28-year-old shepherd man living in rural area of northern part of Afghanistan with chief complaint of cough and shortness of breath for last four years was referred for a chest CT examination. CT images revealed a large, well defined, fluid attenuating, non-enhancing cystic mass lesion in the left lung mostly occupying the left upper lobe measuring approximately 17 × 11 × 15 cm (Fig. 4a,b). Some internal septations were seen in the superior aspect of this cystic lesion (Fig. 4a- arrow). A multiseptated, fluid attenuating cystic lesion was seen in the cardiac apex measuring approximately 6.5 × 5.7 × 5 (Fig. 5). The lesion demonstrated some peripheral wall calcification in pre- contrast images (Fig. 4-curved arrows).

Keeping in mind multiplicity of the lesions, typical CT features, contact with animals and geographic location of the patients, the diagnosis of hydatid cysts was made for both of the patients.

Unfortunately, as the patients were sent to the authors department only for CT examinations, therefore further clinical and laboratory information (including blood tests and ECG findings) as well as follow up of treatment is unavailable.

## Discussion

The first case of cardiac hydatid cyst has been reported by Williams in 1936 [3]. Echinococcosis is a parasitic infection caused by Echinococcus granulosus. Dogs are definitive hosts while humans are accidentally affected. After being eaten by the host, the parasite penetrates in the mucosa of the gastrointestinal system and reaches the portal venous system. Liver acts as first filter for trapping the ova of Echinococcus granulosus while second filter is pulmonary capillary bed. When some of the embryos escape these two filters, they can reach any tissue of the body including the myocardium. Ova can spread to the heart through the coronary circulation, pulmonary veins, intestinal lymphatic vessels, thoracic duct, superior and inferior vena cava, and even hemorrhoidal veins of large intestine [4, 5].

The most common location in the heart is the left ventricle (60%) [as in both of our cases] followed by the right ventricle (15%), the intreventricular septum (9%), the left atrium (8%), the right atrium (4%), and interatrial septum (2%) respectively [6]. The maturation of the cyst can take from one to five years [7].

Early diagnosis of the cardiac hydatid cysts is difficult because of long latency period between exposure to the infection and manifestation of disease. Cardiac cyst can be asymptomatic however it can present with chest pain, dyspnea, palpitations, ventricular tachycardia, fibrillation, cardiac tamponade as well as signs and symptoms related to cardiac chambers outflow obstruction and atrioventricular nodal blocks [7, 8].

Electrocardiographic findings include T-wave inversion and premature ventricular beats [9]. Chest radiograph findings are nonspecific. It can occasionally detect cardiomegaly depending of the site and size of the lesion. Echocardiography is a good diagnostic tool. It can show the size, location, wall calcification and internal septations on the lesion (if present) and also associated findings like pericardial effusion, but in some cases it cannot differentiate between soft tissue mass lesions and hydatid cyst, therefore contrast enhanced CT and cardiac magnetic resonance imaging (MRI) may be needed [10] . Eosinophilia can be detected in hematologic tests; and serological test can be positive in 50% of case [11].

The treatment of cardiac hydatid cyst is surgery [3]. Early surgery is considered safe and has satisfactory result, which prevents from life threatening complications. Gentle manipulation of the heart minimizes the risk of lethal complications, such as rupture and embolization of germinative membrane. The surgeon should be prepared for such possibility and take appropriate steps to prevent contamination of surrounding stricture by the parasite [7, 12].

Drug therapy with Mebenadazole and recently with Albendazole is used, however not as definitive therapy, but to prevent post-operative recurrence [13].

Sudden death after surgery in late period is a rare complication and it is believed to happen because of either rupture of cysts that were not discovered during surgery or secondary cysts development as a result of leakage from the primary cyst [14].

## Conclusion

Cardiac hydatid cyst is a rare finding with wide range of signs and symptoms. Cardiac hydatid cysts can be suspected in patients coming from endemic areas. Echocardiographic follow up of patients with liver or lung hydatid cysts can be helpful.

## Abbreviations

CT: computed tomography
MRI: Magnetic resonance imaging
